# Structural Characteristics, Antioxidant and Hypoglycemic Activities of Polysaccharide from *Siraitia grosvenorii*

**DOI:** 10.3390/molecules27134192

**Published:** 2022-06-29

**Authors:** Pin Gong, Yuxi Guo, Xuefeng Chen, Dandan Cui, Mengrao Wang, Wenjuan Yang, Fuxin Chen

**Affiliations:** 1 School of Food and Biological Engineering, Shaanxi University of Science and Technology, Xi’an 710021, China; guoyuxi416@163.com (Y.G.); chenxf@sust.edu.cn (X.C.); dyx1210842405@163.com (D.C.); 13488311504@163.com (M.W.); yangwenjuan@sust.edu.cn (W.Y.); 2 School of Chemistry and Chemical Engineering, Xi’an University of Science and Technology, Xi’an 710054, China; chenfuxin1981@163.com

**Keywords:** *Siraitia grosvenorii* polysaccharides, structure characterization, hypoglycemic activity, antioxidant activity, structure–function relationship

## Abstract

The structural characterization, the in vitro antioxidant activity, and the hypoglycemic activity of a polysaccharide (SGP-1-1) isolated from *Siraitia grosvenorii* (SG) were studied in this paper. SGP-1-1, whose molecular weight is 19.037 kDa, consisted of Gal:Man:Glc in the molar ratio of 1:2.56:4.90. According to the results of methylation analysis, GC–MS, and NMR, HSQC was interpreted as a glucomannan with a backbone composed of 4)-*β*-D-Glc*p*-(1→4)-, *α*-D-Glc*p*-(1→4)-, and 4)-Man*p*-(1 residues. *α*-1,6 linked an *α*-D-Gal*p* branch, and *α*-1,6 linked an *α*-D-Glc*p* branch. The study indirectly showed that SGP-1-1 has good in vitro hypoglycemic and antioxidant activities and that these activities may be related to the fact that the SGP-1-1’s monosaccharide composition (a higher proportion of Gal and Man) is the glycosidic-bond type (*α*- and *β*-glycosidic bonds). SGP-1-1 could be used as a potential antioxidant and hypoglycemic candidate for functional and nutritional food applications.

## 1. Introduction

In recent years, the number of people with diabetes mellitus has increased dramatically due to people’s irrational diets and lifestyles. There are 425 million people with diabetes worldwide, which means that one in every 11 people in the world has diabetes [1]. However, most of the drugs currently used to treat diabetes have some side effects. Therefore, the development of a natural drug with fewer side effects has become a major point of current diabetes mellitus research [2]. In recent years, several plant polysaccharides have been shown to exhibit improved hypoglycemic activity and can improve insulin resistance (IR) with low toxicity and side effects [3,4,5,6,7,8,9,10].

*Siraitia grosvenorii* (SG) is the perennial fruit of a vine in the *Cucurbitaceae* family, originally grown in southern China, especially in Guangxi [11]. SG has the characteristics of a sweet taste being low-calorie, as well as unique benefits related to the elimination of throat inflammation, moistening the lung for phlegm removal, and the regulation of intestinal and liver function, which are mainly due to many bioactive components in SG [12]. It is noteworthy that studies related to the bioactive components in SG have focused on flavonoids and triterpenoids [11], but in-depth studies on the structure, efficacy, and constitutive relationships of *Siraitia grosvenorii* polysaccharides (SGPs) are still needed [9,10,11,12]. Moreover, Zhu et al. showed that SGP has some antioxidant effects in vitro, especially on the scavenging of DPPH [13]. In addition, SGP also concentration-dependently reduced the overproduction of ROS and apoptotic/necrotic cells in H_2_O_2_-injured PC1_2_ cells. Previous studies by our group reported that the inflammatory and antioxidant effects of SGP-1-1 (a polysaccharide extracted from SG) were studied in an inflammation-suppressed mouse model of diabetic nephropathy and elucidated the underlying molecular mechanisms of inflammation and oxidative stress in SGP-1-1 through a treated mouse model [14]. However, the specific conformational relationship between the hypoglycemic activity and the structure of SGP-1-1 is unknown.

In this paper, a polysaccharide from dried fruit of SG was isolated (named SGP-1-1) and purified, which emphasized the chemical composition and the structural and physical characteristics. This was performed to further the structural–functional relationships of SGP-1-1 and to expand the application of SG resources. Our study will provide a data basis for the wide utilization of SG and its active components, which could be used as a potential antioxidant and hypoglycemic candidate for functional and nutritional food applications.

## 2. Materials and Methods

### 2.1. Materials and Reagents

Dried SG was purchased in Xi’an City, Shanxi Province, China. _L_-Butanol, 3,5-dinitrosalicylic acid (DNS), _D_-glucuronide, pyridine, crystalline bovine serum protein, DEAE cellulose, *α*-glucosidase, acarbose, 4-nitrophenyl-*α*-_D_-glucopyranose (PNPG), _D_-rhamnose, _L_-arabinose, _D_-mannose, _D_-fucose, _D_-galactose, galacturonic acid, glucuronic acid, and _D_-ribose were purchased from Shanghai Genye, China. Metformin (H20023370, China) was purchased from Tong Ren Tang Pharmacy, Xi’an, Shanxi, China.

### 2.2. Isolation and Purification of SGP-1-1

Dried SG was crushed, sieved, degreased in ethanol for 48 h, oven-dried, and set aside. The extraction was carried out in hot water at 70 °C for 2.5 h at a liquid to the material ratio of 1:35 g/mL. Proteins were removed by the Sevag method [8]. The extracts were concentrated and precipitated in ethanol at 4 °C for 24 h. The precipitate was then centrifuged and re-solubilized with water. Finally, the crude polysaccharide was freeze-dried and subsequently isolated and purified.

**Figure 1:**
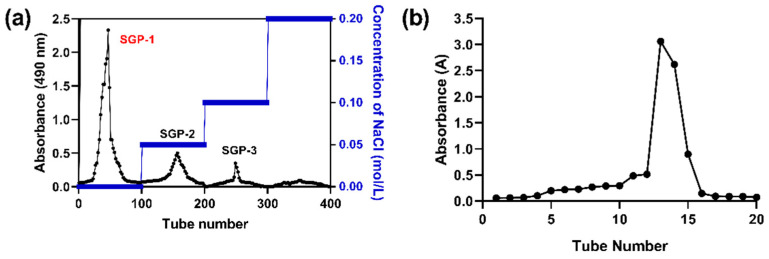

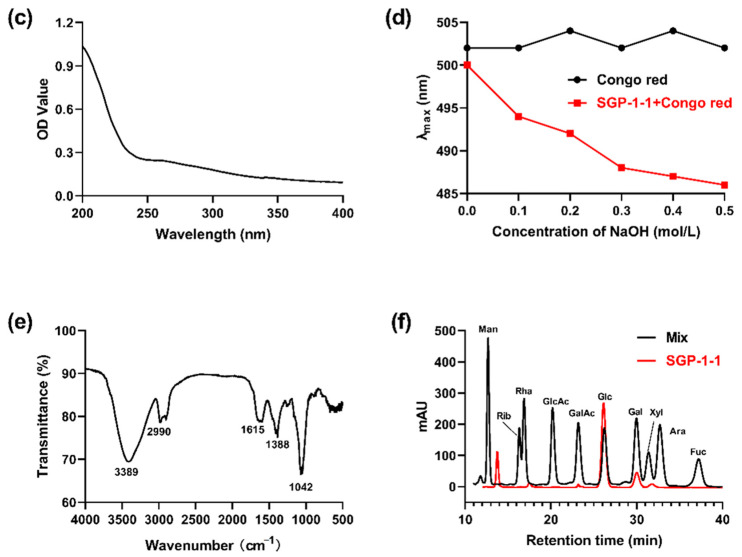
(**a**) Chromatography by DEAE Sepharose Fast Flow; (**b**) Sephadex G 100 separation; (**c**) UV spectra; (**d**) triple-helix-structure analysis; (**e**) FT-IR spectrum; (**f**) monosaccharide-composition analysis of SGP-1-1.

The SGP was dissolved in distilled water to prepare a sample solution of 10 mg/mL and loaded onto an ion-exchange column of DEAE Cellulose-52, which was subjected to a gradient elution using distilled water, 0.05, 0.10, 0.15, and 0.20 mol/L NaCl solution at a flow rate of 1 mL/min, respectively. Next, the purified fraction was collected and concentrated, and then dialyzed for 48 h to eliminate NaCl. Three fractions (SGP-1, SGP-2, and SGP-3) were obtained (Figure 1a). We pre-assayed the in vitro antioxidant activity of these three polysaccharides, with SGP-1 showing the strongest activity, for subsequent purification to continue other in-depth experiments.

The main fraction obtained (SGP-1) was then further purified by Sephadex G-100 gel-permeation chromatography (Φ20 mm × 600 mm) eluting with ultrapure water at a flow rate of 0.5 mL/min. After concentration and freeze-drying, the purified polysaccharide was obtained (named SGP-1-1). A polysaccharide fraction SGP-1-1 was finally yielded and concentrated for further study (Figure 1b).

### 2.3. Determination of SGP-1-1 Total Sugar, Protein, and Reducing Sugar Content

The total sugar content was measured by the phenol-sulfuric-acid method [12]. The protein content was determined by the Bradford method [13]. Reducing sugar content was assayed by the DNS method [15].

### 2.4. Structural Characterization of SGP-1-1

#### 2.4.1. Molecular Weight (Mw)-Determination Analysis

The *Mw* distribution was measured by gel-permeation chromatography (GPC), a system equipped with a refined index detector and an Agilent PL aquagel-OH MIXED-H column (300 mm × 7.5 mm, 8 µm). The SGP-1-1 sample (5.0 mg/mL, 20 μL) was purified by 0.1 M NaNO_3_ at a stream rate of 0.6 mL/min and a temperature of 35 °C. Molecular weights of the fractions were calculated by constructing calibration curves.

#### 2.4.2. Monosaccharide-Composition Analysis

The composition of the monosaccharides was analyzed by modified pre-column PMP derivatives of carbohydrates [16]. Briefly, SGP-1-1 (2 mg) was directly hydrolyzed with 2 mmol/L trifluoroacetic acid (TFA) at 120 °C for 2 h. After removal of TFA under nitrogen, the hydrolysate was subsequently dried under vacuum and subjected to derivatization with 50 μL of 0.3 mmol/L NaOH and 50 μL of PMP (0.5 mmol/L, methanol) at 70 °C for 60 min. The reaction was neutralized with 50 μL of 0.3 mmol/L hydrochloric acid to stop the reaction, diluted with 1 mL of 0.1 mmol/L sodium phosphate, and extracted 3 times with 1 mL of chloroform. The water layer is ultrafiltered through a 0.22 µm film for HPLC analysis on an Agilent 1200 series HPLC system configured with a counter-phase Ultimate C18 column (4.6 mm × 250 mm, 5 µm). UV detection was conducted at 248 nm. The mobile phase comprised 83% (*v*/*v*) sodium phosphate (0.1 mmol/L, pH 6.9) and 17% (*v*/*v*) acetonitrile, which eluted at 30 °C at a flow rate of 1.0 mL/min. The monosaccharide composition was identified, quantified based on its retention time and integral area, and compared with existing commercial standards.

#### 2.4.3. Ultraviolet (UV) and Fourier Transform Infrared (FT-IR)-Spectra Analysis

A 0.1 mg/mL solution of SGP-1-1 polysaccharide was prepared and analyzed using UV at 200–400 nm with a time interval of 10 nm (Thermo Fisher Scientific Co., Waltham, MA, USA) [17]. The polysaccharide sample was scanned in a FT-IR spectrometer at the range 4000–400 cm^−1^ (Bruker, Billerica, Germany).

#### 2.4.4. Nuclear Magnetic Resonance (NMR)-Spectroscopy Analysis

The structure of SGP-1-1 was analyzed by NMR spectroscopy (Bruker, Billerica, Germany) [18]. Briefly, 40 mg of dried polysaccharide sample was dissolved in D_2_O) SGP-1-1 was then analyzed by ^1^H NMR, ^13^C NMR, DEPT-135 NMR, homonuclear ^1^H-^1^H correlation spectroscopy (COSY), and heteronuclear single-quantum-correlation spectroscopy (HSQC) using an Avance 800 NMR spectrometer (Bruker) at 25 °C. The scanning number for^1^H NMR was 4, while the scanning number of ^13^C and DEPT-135 NMR was 96, respectively. The scanning numbers of COSY and HSQC, were 2 and 16, respectively.

#### 2.4.5. Congo-Red Test

The Congo-red test was performed referring to the method of Liu et al. with minor modifications [19]. Briefly, 0.5 mg/mL of SGP-1-1 solution was prepared, so 2 mL of SGP-1-1 solution and an equal volume of 50 μM of Congo red solution were added to each tube, and then 0.1, 0.2, 0.3, 0.4, and 0.5 M of NaOH solution were added, sequentially, and left to stand 30 min. The maximum absorption wavelength (*λ*_max_) of each tube was recorded in the range of 400–600 nm at 25 °C, in parallel three times in sequence.

#### 2.4.6. Morphology Analysis

The morphology of SGP-1-1 was analyzed by SEM. The dried-polysaccharide fraction was sputtered with gold powder, and its surface features and microstructure were investigated by SEM (JMS6700 F, JEOL Co., Tokyo, Japan) at an accelerating voltage of 8 kV.

#### 2.4.7. Methylation Analysis

Methylation analysis of SGP-1-1 was performed according to the method reported by Zhang et al. [20]. The methylated polysaccharide was hydrolyzed with 2 mmol/L TFA at 120 °C for 2 h, followed by acetylation with NaBD_4_ and acetic anhydride to give the methylated-alcohol acetate. The methylated-alditol acetate was analyzed by GC-MS using an HP-5MS fused-silica capillary (30 cm × 0.25 mm, 0.25 μm) and a flame-ionization detector (FID) with helium as the carrier gas. The temperature program was as follows: 80 °C for 1 min, 10 °C/min, 80 °C/min, held for 10 min, 160–200 °C at 2 °C/min, and then held for 20 min. The syringes and detectors were heated at 250 °C and 280 °C, respectively. The interpretation of the mass spectra obtained led to the identification of the compound corresponding to each peak. The peak area was utilized to obtain the molar ratio for each residue.

### 2.5. In Vitro Antioxidant Capacity

DPPH-scavenging activity was determined following a reported method [21]. OH-scavenging rate was analyzed according to the method of Fenton reaction [21]. ABTS^+^-scavenging activity was evaluated according to the reported method [21].

### 2.6. In Vitro Hypoglycemic Activity

#### 2.6.1. In Vitro α-Glucosidase- and α-Amylase-Inhibitory-Activity Assay

In vitro *α*-glucosidase-inhibitory activity was established according to the previous method with minor modification [5]. In vitro *α*-amylase-inhibitory activity was measured following a reported method [15].

#### 2.6.2. Construction of an Insulin Resistance (IR)-HepG2 Model

The effect of insulin concentration on cell viability was examined using the MTT method [21]. Appropriate amounts of log-phase cells were taken, washed 2–3 times with PBS buffer, and then digested by adding trypsin. The medium was terminated, the supernatant was discarded by centrifugation, and 1 mL of complete medium was added and gently blown through. The cell suspension and TissueBlue staining solution were mixed thoroughly by 1:1 aspiration into a 1.5 mL centrifuge tube, counted on a cell counting plate, and diluted with complete medium after calculation. The cell density was adjusted to 5 × 10^4^/well and the cells were inoculated in 96-well plates overnight at 100 μL per well, with blank, control, and insulin groups set up. After the cells were plastered, the old medium was discarded, and serum-free medium was added and starved for 10 h. After that, serum-free medium containing 10^−9^, 10^−8^, 10^−7^, 10^−6^ and 10^−5^ mol/L insulin was added at 10 μL per well, with 6 replicate wells for each concentration. The 96-well plate was incubated in the incubator for 12 h. Afterwards, 10 μL of MTT solution was added to each well of the plate and incubated in the incubator for 4 h. The plate was gently shaken, and the absorbance values were measured at 450 nm.

#### 2.6.3. Assay of Hexokinase (HK), Pyruvate Kinase (PK) Activities, Triglyceride (TG), and Glycogen Content in HepG2 Cells

Logarithmic-growth-stage HepG2 cells were collected precisely, trypsinized, and cell-counted. The cell density was adjusted to 2 × 10^5^/well and inoculated in 96-well plates. The blank group was the medium group without cells, the positive control group was the metformin-treated group, and the drug group was added to complete cultures of SGP-1-1 at low, medium, and high doses of 10, 40, and 160 μg/mL, respectively. HK, PK activities, TG, and glycogen content were determined using a glucose-assay kit (Nanjing Jiancheng Institute of Biological Engineering, Nanjing, China) in accordance with the instructions of the manufacturer.

### 2.7. Statistical Analysis

Results were expressed as mean ± SD, and differences between groups were evaluated by one-way analysis of variance, followed by the least-significant-difference post hoc test. *p* values less than 0.05 were considered statistically significant. All statistical analyses were performed using SPSS Statistics for Windows, version 20.0 (SPSS Inc., Chicago, IL, USA).

## 3. Results and Discussion

### 3.1. Total Polysaccharide, Reducing Sugar, and Protein Content of SGP-1-1

The total polysaccharide content of SGP-1-1 measured in this study was 87.34%, the reducing sugar content was 2.06%, and the protein content was 0.45%.

### 3.2. Structural Characterization of SGP-1-1

#### 3.2.1. Mw and Monosaccharides Composition of SGP-1-1

The *Mw* of the sample is calculated as 19.037 kDa. SGP-1-1 had a monosaccharide composition of Galactose (Gal), Mannose (Man), and Glucose (Glc) in the molar ratio of 1:2.56:4.90 (Figure 1f). By this result, Zhu et al. [10] reported that SGP residues were composed of Ara, Man, Glc, Gal, GlcAc, and GalAc, with the ratio of 1:1.92:3.98:7.63:1.85:7.34, which may be due to the different sources of SG (fresh fruit). This is also confirmed by a study by Yan et al. [22], that drying has a relatively large effect on the monosaccharide composition of plant fruit (*Momordica charantia* L.) polysaccharides. This may be because drying alters the microstructure of the polysaccharide.

#### 3.2.2. Spectroscopic Characteristics of SGP-1-1

As indicated in Figure 1c, the UV scan showed no significant absorption peaks at 260 nm and 280 nm, which means that the protein and nucleic acid were eliminated. The FT-IR spectrum of SGP-1-1 is shown in Figure 1e. The FT-IR spectrum of SGP-1-1 displayed a broad stretching peak at 3389 cm^−1^ and a weak absorption at 2990 cm^−1^ and 1388 cm^−1^ attributed to the typical O-H and C-H stretching vibration of the polysaccharide, respectively [17,20]. The peak at 1615 cm^−1^ further showed the presence of C=O of the carboxylate group, which agreed with the result of the meta-hydroxy-diphenyl method [23]. The absorptions between 1000 and 1200 cm^−1^ were assigned to a pyranose form of sugars [24], and the peak at 760 cm^−1^ indicated the presence of *β*-type glycosidic linkages in SGP-1-1 [21].

#### 3.2.3. Methylation Analysis

The GC-MS analysis of the hydrolysate of methylated SGP-1-1 was shown in Table 1, Figure S1. The high proportion of the molecular fragments were 2,3,4,6-*O*-Me_4_-Glc*p*, 2,3,4-*O*-Me_3_-Glc*p*, and 2,3,4,6-*O*-Me_4_-Man*p*. This indicated that 1,4-linked-Man*p* and 1,6-linked-Glc*p* were the components of the backbone. That also means that the branched chain of SGP-1-1 was made up of Gal and linked with 1→6 glycosidic bonds at six positions. This result is consistent with the findings of the monosaccharide-composition-ratio analysis.

#### 3.2.4. NMR Spectra of SGP-1-1

In the H-spectrum analysis (Figure S2a), when the isotopic hydrogen-proton signals of polysaccharides are mostly in the range of δ3.5 ppm to 5.5 ppm, the signals of *α*-constituent polysaccharides appear in the range of δ4.8 ppm to 5.3 ppm, and the signals of *β*-constituent polysaccharides appear in the range of δ4.4 ppm to 4.8 ppm. SGP-1-1 has two heterotrimeric-hydrogen signals between δ4.3 ppm and δ5.02 ppm, indicating that the polysaccharide SGP-1-1 has both *α*- and *β*-glycosidic bonds. NMR C-spectroscopy analysis (Figure S2b) showed that the signals of C3 and C5 appeared in the range of δ82 ppm to 88 ppm for the furan ring and less than δ80 ppm for the pyran ring. SGP-1-1 contains both *α*- and *β*-glycosidic bonds.

**Figure 2:**
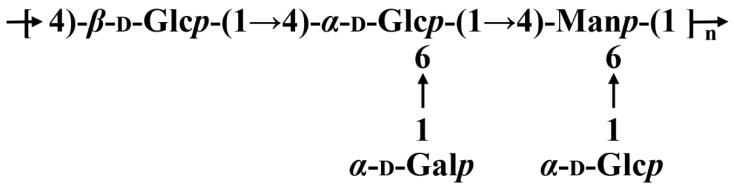
Predicted backbone structure of SGP-1-1.

To further illustrate the skeletal structure of SGP-1-1, ^1^H- and ^13^C-NMR signals are according to HSQC, and ^1^H-^1^H COSY is comfortable with spectral-correlation assignments (Figure S2c,d). Although not all NMR signals can be recognized because of the complexity of the spectra, a few characteristic peaks are listed in Table 2. At δ 4.3–5.02 ppm, chemical shifts in the ^1^H-NMR spectrum at δ 82–170 ppm in the ^13^C-NMR spectrum (Figure S2) were assigned to the anomalous-proton signals and silicon–carbon signals of the glycosyl residues, indicating that the polysaccharide has a very complex structure. Considering the many signals in the anomalous region, as shown in Table 2, the glycosyl residues are designated as A-D for clarity of description. Based on 2D COSY NMR analysis and references, the chemical shifts attributed to the sugar residues in the sugar chain are shown in Table 2, from which the sugar residues contained in SGP-1-1 are →4)-*α*-_D_-Glc*p*→1, →6)-*α*-_D_-Glc*p*-(1→, →4,6)-*α*-_D_-Man*p*-(1→, and *β*-_D_-Glc*p*-(1→. Combined with the analysis of methylation and the ratio of monosaccharide composition, the structure of SGP-1-1 is shown in Figure 2.

#### 3.2.5. Congo-Red Test of SGP-1-1

Hydrogen bonding is the primary molecular force in the helical conformation, and the triple-helix structure of polysaccharides is sustained by hydrogen bonds [23]. The changes of the maximum-absorption wavelength of SGP-1-1, complexed with Congo red in different concentrations of NaOH solution, are shown in Figure 1d. The maximum-absorption wavelength did not change with the increase in NaOH concentration and was always between 500–504 nm, indicating that the polysaccharide sample did not undergo deconvolution and did not have a three-stranded helical structure.

#### 3.2.6. Microstructure of SGP-1-1

**Figure 3:**
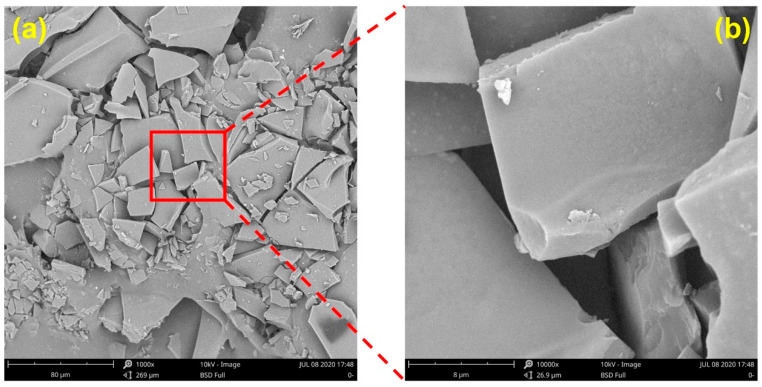
Morphology analysis of SGP-1-1 by SEM. (**a**) 1000-fold; (**b**) 10,000-fold.

The microscopic structure of SGP-1-1 is shown in Figure 3. Irregular shapes exist and are easy to aggregate into clusters. It can be assumed that the intermolecular forces of polysaccharides are large and diverse, which is one of the properties of polysaccharides [25,26,27]. In addition, probably due to the low magnification, the SEM image of SGP (500×) reported by Zhu et al. [13] showed a flocculent, interwoven structure.

### 3.3. Antioxidant Activities In Vitro

**Figure 4:**
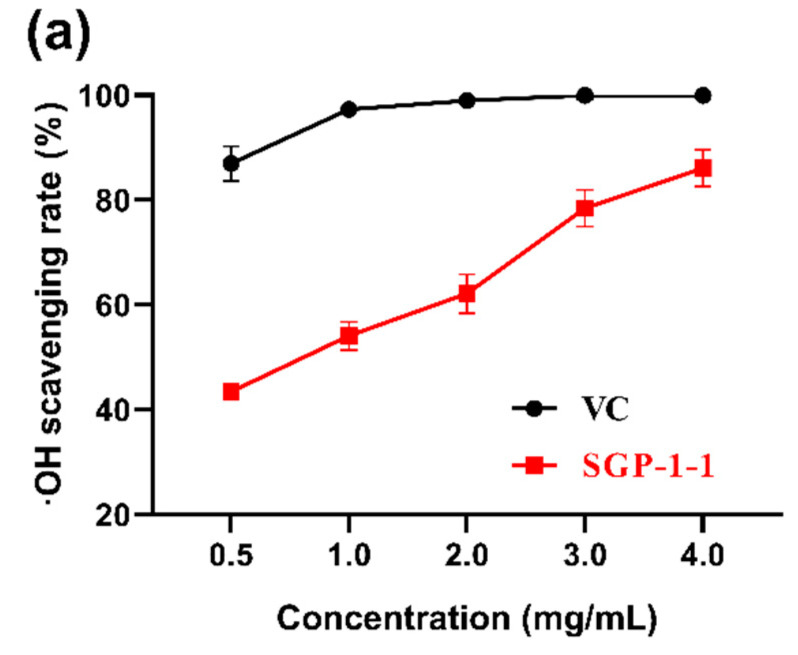

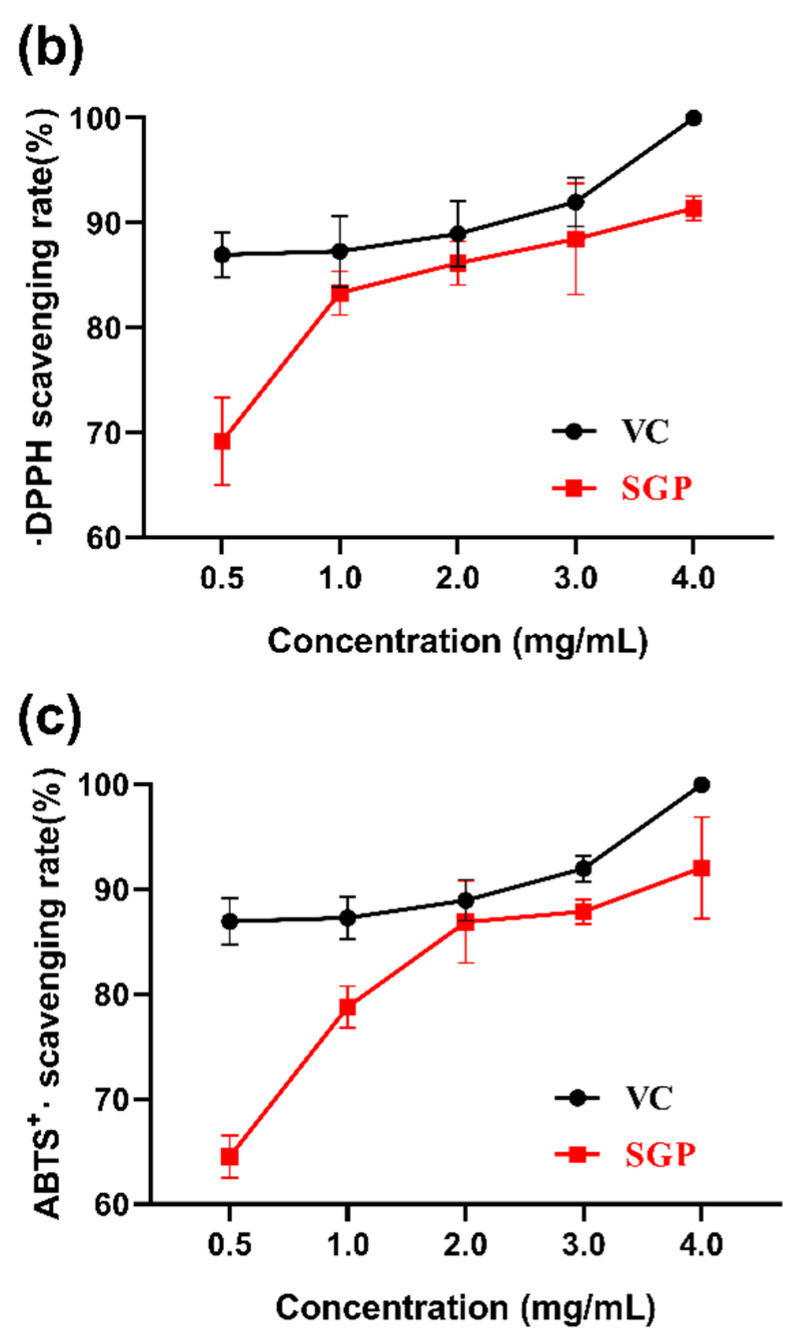
In vitro free-radical-scavenging abilities of SGP-1-1. (**a**) DPPH·-scavenging ability of SGP-1-1; (**b**) ·OH-scavenging ability of SGP-1-1; (**c**) ABTS^+^·-scavenging ability of SGP-1-1.

The ·OH-scavenging rates of SGP-1-1 were illustrated in Figure 4a. With the increase in SGP-1-1 concentration, the OH-scavenging rate showed an increasing trend, which was consistent with the results of the ·OH-scavenging rate of most plant polysaccharides [23,28,29]. The scavenging effect of SGP-1-1 on ·OH-scavenging rates increased in a concentration-dependent manner. The OH-scavenging rate of SGP-1-1 in this study was as high as 81.38%. The DPPH·-scavenging rates of SGP-1-1 were demonstrated in Figure 4b, where the scavenging rates of DPPH showed an increasing trend with increasing sugar concentration. This is consistent with the findings of most polysaccharide extracts [30,31,32,33]. The DPPH·-scavenging activity of SGP-1-1 reached 89.12%. The scavenging rates of ABTS·^+^ by SGP-1-1 are shown in Figure 4c. With the rose of sugar concentration, the scavenging rate of ABTS^+^·showed an increasing trend. At a concentration of 4 mg/mL, the ABTS^+^·-scavenging activity of SGP-1-1 reached 88.04%. In most studies, the radical-scavenging rate of ABTS^+^· by the polysaccharides was lower than that of the positive-control compound (Vitamin C, V_C_). However, the scavenging ability of polysaccharide fractions from *Citrus aurantium*, *geranium,* and *ginseng* on ABTS·^+^ was reported to be comparable to, or even stronger than, that of V_C_ [34,35]. The scavenging of DPPH, ABTS^+^, and OH by quinoa polysaccharide at 4 mg/mL was 21.71 ± 1.25%, 49.46 ± 1.59%, and 70.10 ± 3.39%, respectively [36]. *Codonopsis tangshen Oliv* polysaccharide has strong DPPH·-scavenging activity (IC_50_ of 0.610 mg/mL) [37].

### 3.4. Hypoglycemic Activities of SGP-1-1

#### 3.4.1. In Vitro Inhibition of α-Glucosidase and α-Amylase Activities

**Figure 5:**
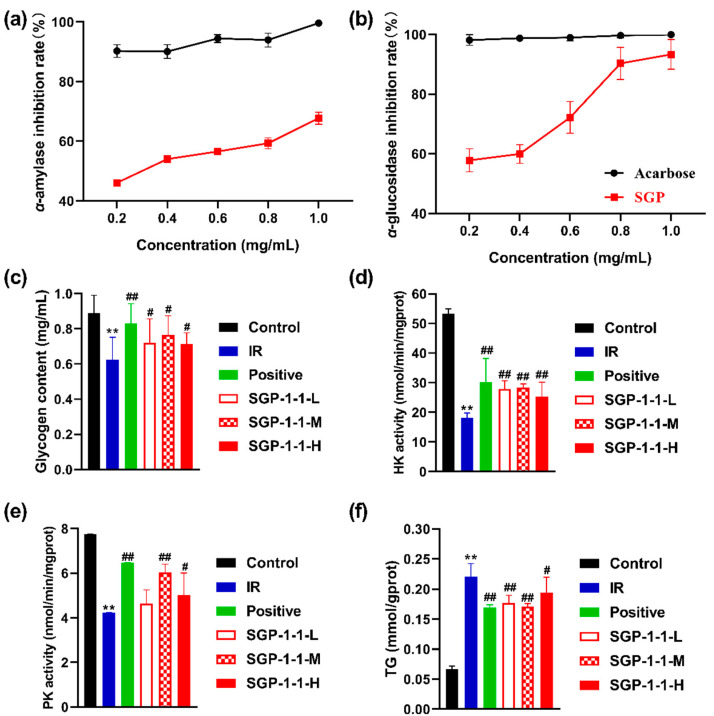
In vitro hypoglycemic activities of SGP-1-1. (**a**) Inhibition of α-amylase activities; (**b**) inhibition of α-glucosidase activities; (**c**) glycogen content of IR-HepG2 cells; (**d**) hexokinase (HK) and (**e**) pyruvate kinase (PK) activities of IR-HepG2 cells. (**f**) TG content of IR-HepG2 cells; compared to Control group, ** *p* < 0.001; compared to IR group, # *p* < 0.01, ## *p* < 0.001 (IR, insulin resistance; Positive, metformin-treated group).

*α*-glucosidase has a hydrolytic role in glucose-catalyzed reactions, breaking the *α*-1,4 glycosidic bond at the non-reducing end of *α*-glucoside, oligosaccharide, and glucan to release glucose. When the *α*-glucosidase activity decreases, the postprandial-blood glucose levels fall, so inhibition of *α*-glucosidase activity regulates glycaemia [38]. The inhibitory activities of SGP-1-1 on *α*-amylase are shown in Figure 5a. The inhibitory activity of *α*-amylase increased with the increase in polysaccharide concentration, and the inhibition rate of *α*-amylase showed an increasing trend. At the sample concentration of 1 mg/mL, SGP-1-1 showed the best inhibition of *α*-amylase with 61.73%. SGP-1-1 inhibited *α*-glucosidase activity, as shown in Figure 5b; the inhibition of *α*-glucosidase showed an increasing trend with the increase in sugar concentration. At the sample concentration of 1 mg/mL, SGP-1-1 showed the best inhibition of *α*-glucosidase with 93.34%. Polysaccharides isolated from mulberry fruits [39], *Inonotus obliquus* [40], and *Sargassum thunbergii* [41] possess both antioxidant and *α*-glucosidase-inhibiting abilities. The inhibitory activity of licorice polysaccharide against *α*-glucosidase showed that the inhibition increased with increasing concentration. When the concentration reached 6 mg/mL, the inhibition of α-glucosidase activity reached 64.77% [5]. Red-clover polysaccharide showed 86.72% inhibition of *α*-glucosidase at an assay concentration of 10 mg/mL compared to acarbose [3].

#### 3.4.2. Glycogen Content of IR-HepG2 Cells

Glycogen is a multi-branched glucose polysaccharide that is used in the body as a form of energy storage [42,43]. The glycogen content of SGP-1-1 on IR-HepG2 cells is shown in Figure 5c, where the IR group showed a significant decrease (*p* < 0.01) in HepG2-cell glycogen content compared to the control group, which was approximately 70.31% of the control group. Compared to the IR group, the positive-control group and the SGP-1-1 low-, medium-, and high-dose groups showed a significant increase in TG content (*p* < 0.01). This agreed with the findings of Cao et al. on the hypoglycemic activity of *Sargassum plumosa* polysaccharide (PSP-1) in vitro [44]. This suggested that all doses of SGP-1-1 treatment can increase the glycogen content of IR-HepG2 cells.

#### 3.4.3. Hexokinase (HK) and Pyruvate Kinase (PK) Activities of IR-HepG2 Cells

Insulin resistance usually results in reduced glucose utilization and decreased regulation of hepatic-glycolytic enzymes such as hexokinase (HK) and pyruvate kinase (PK) [15]. To investigate the effects of SGP-1-1 on the regulation of two key liver enzymes in glucose metabolism, HK and PK were treated with different concentrations of SGP-1-1. As shown in Figure 5d,e, IR may lead to reduced glucose utilization and down-regulation of hepatic-glycolytic-enzyme expression, such as HK and PK, and the SGP-1-1 concentration groups showed a significant increase in HK and PK activity compared to the IR group (*p* < 0.05). The HK and PK activities of the medium-dose treated group were close to those of the positive group. The same trend was reported in previous studies for *Sarcandra glabra* polysaccharides [45] and *Sargassum plumosa* polysaccharides [46]. In conclusion, SGP-1-1 has a considerable effect on glucose metabolism by regulating the activity of key related enzymes.

#### 3.4.4. TG Content of IR-HepG2 Cells

The TG content of SGP-1-1 on IR HepG2 cells was shown in Figure 5f. As compared to the control group, the TG content of HepG2 cells was significantly higher in the IR group (*p* < 0.01), which was about 3.3-fold higher than that of the control group. Compared with the IR group, the TG content of the positive control and the SGP-1-1 low-, medium-, and high-dose groups was significantly lower (*p* < 0.01), with 23.19%, 19.6%, 22.36%, and 11.75%, respectively. This implies that all doses of SGP-1-1 treatment improved the TG content of IR-HepG2 cells.

### 3.5. Relationship between Structure and Biological Activity

**Figure 6:**
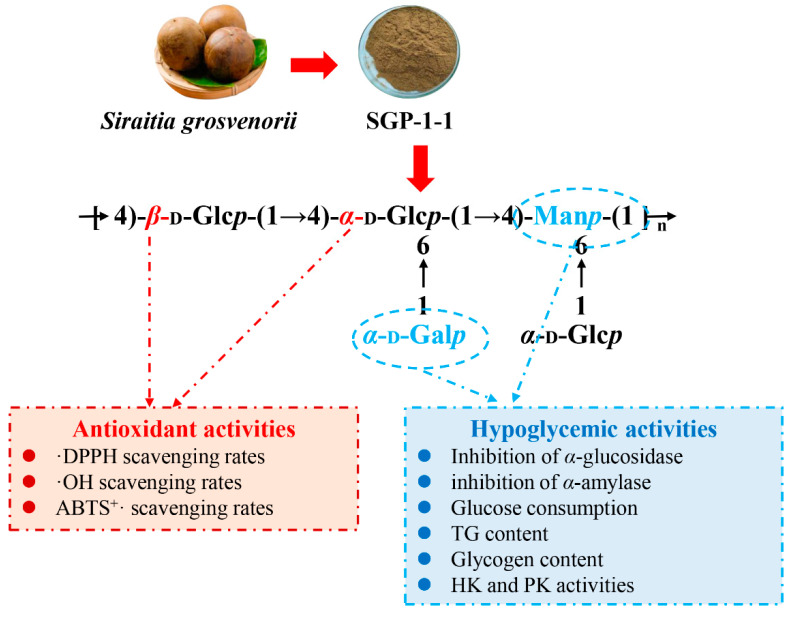
Relationship between structure and biological activity of SGP-1-1.

The antioxidant and hypoglycemic activity of SGP-1-1 is closely related to its monosaccharide composition (a higher proportion of Gal and Man), glycosidic-bond type (contains both *α*- and *β*-glycosidic bonds) and lack of three-stranded helical structure (Figure 6). The monosaccharide composition of SGP-1-1 contains a high proportion of Man and Gal, which may be related to its hypoglycemic activity [11]. Cao et al. obtained a polysaccharide with high Man content (PSP-1) from *Sargassum pallidum*. The results of hypoglycemic experiments showed that PSP-1 had good *α*-amylase- and *α*-glucosidase-inhibitory activities and significantly promoted glucose consumption, glycogen synthesis, and PK and HK activities in insulin-resistant cells [41]. Microwave-assisted extraction of *S. cerevisiae* polysaccharide (STP-1) also has a high content of Man, possesses strong antioxidant- and *α*-glucosidase-inhibitory activity, and improves glucose uptake by IR-HepG2 cells [41]. Moreover, SGP-1-1 contains both *α*- and *β*-glycosidic bonds, which may contribute to its superior in vitro antioxidant and hypoglycemic activity. The major glycosidic bond of the polysaccharide MFP-2A isolated from *Mallotus furetanus* (*Baal*) *Muell Arg* consists of both *α*- and *β*-glycosidic bonds. In contrast, in previous studies, MFP-2A also showed remarkable free-radical-scavenging ability and *α*-glucosidase-inhibitory activity in vitro [43]. Notably, polysaccharides with hypoglycemic activity do not apparently have a three-stranded helical structure [41]. SGP-1-1, without a three-stranded helical structure, exerts excellent hypoglycemic activity by in vitro assay and cell experiment. Jia et al. studied an ultrasound-assisted enzymatic extraction of two polysaccharides (SFP-1 and SFP-2) from *Sargassum fusiforme*, which do not have a triple-helix structure but had significant hypoglycemic activity [4].

## 4. Conclusions

In conclusion, this study elucidated the structure of SGP-1-1 from SG, as well as its antioxidant activity and hypoglycemic effect in vitro. The results showed that SGP-1-1 (*Mw* = 19.037 kDa) consists of Gal:Man:Glc in the molar ratio of 1:2.56:4.90. NMR revealed that the polysaccharide of SGP-1-1 contains *α*- and *β*-glycosidic bonds and was elucidated as a glucomannan. It does not have a three-dimensional helical structure. In addition, in vitro experiments showed that SGP-1-1 has good antioxidant and hypoglycemic activity. The relationship between the structure and function of SGP-1-1 was also focused on. These results suggest that SGP-1-1 could be used as a potential antioxidant and hypoglycemic component in functional foods.

## Figures and Tables

**Table 1:** Chemical shift assignments of SGP-1-1 by methylation analysis.

Peak No.	Sugar Derivatives	Deduced Residues	Mol%	Mass Fragments (*m*/*z*)
1	2,3,4,6-*O*-Me_4_-Glc*p*	T-Glc*p*	18.4	55, 67, 79, 97, 110, 122, 136, 150, 164, 178, 192, 206
2	2,3,4,6-*O*-Me_4_-Man*p*	1,4-linked-Man*p*	30.4	60, 79, 98, 115, 129, 145, 169, 183, 197, 212, 226, 243
3	2,3,4-*O*-Me_3_-Gal*p*	1,6-linked-Gal*p*	10.5	60, 79, 98, 115, 145, 169, 187, 206, 226, 243, 270
4	2,3,4-*O*-Me_3_-Glc*p*	1,6-linked-Glc*p*	40.7	58, 69, 79, 88, 100, 114, 130, 145, 159, 169

**Table 2:** ^1^H and ^13^C chemical shifts from identified 2D NMR spectra of SGP-1-1.

Sugar Residues		Chemical Shifts (ppm)					
		C1/H1	C2/H2	C3/H3	C4/H4	C5/H5	C6/H6
A	→4)-α-D-Glc*p*→1	5.36	3.57	3.77	3.88	4.01	3.62
		92.18	73.32	72.88	76.89	71.2	61.28
B	→6)-α-D-Gal*p*-(1→	5.17	3.48	3.18	3.49	3.65	3.91
		92.09	71.4	74.12	71.2	73.1	65.4
C	→4,6)-α-D-Man*p*-(1→	5.16	3.57	3.35	4.1	3.82	3.77
		100.63	70.37	71.2	76.23	60.79	66.9
D	*β*-D-Glc*p*-(1→	4.58	4.06	3.43	3.6	3.41	3.72
		95.85	71.1	75.78	73.3	74.1	67.55

## Data Availability

Not applicable.

## Supplementary Materials

The following supporting information can be downloaded at: https://www.mdpi.com/article/10.3390/molecules27134192/s1, Figure S1: GC–MS profile of partially methylated alditol acetates of SGP-1-1 (a) TIC profile and (b-e) MS fragments and deduced residues; Figure S2: NMR spectrum of SGP-1-1 (a.^1^H b.^13^C c. ^1^H- ^1^H COSY d. ^1^C- ^1^HHSQC).
