# Supplementary material for: Structural Characteristics, Antioxidant and Hypoglycemic Activities of Polysaccharide from Siraitia grosvenorii
Source: Molecules. 2022 Jun 29;27(13):4192. doi: 10.3390/molecules27134192 (PMC9268605; doi:10.3390/molecules27134192)
Supplement: Supplementary file 1 [file molecules-27-04192-s001.zip › molecules-1779020-SI.pdf]

Supplementary materials

Figure S1. GC–MS profile of partially methylated alditol acetates of SGP-1-1

(a) TI[illegible]idues.

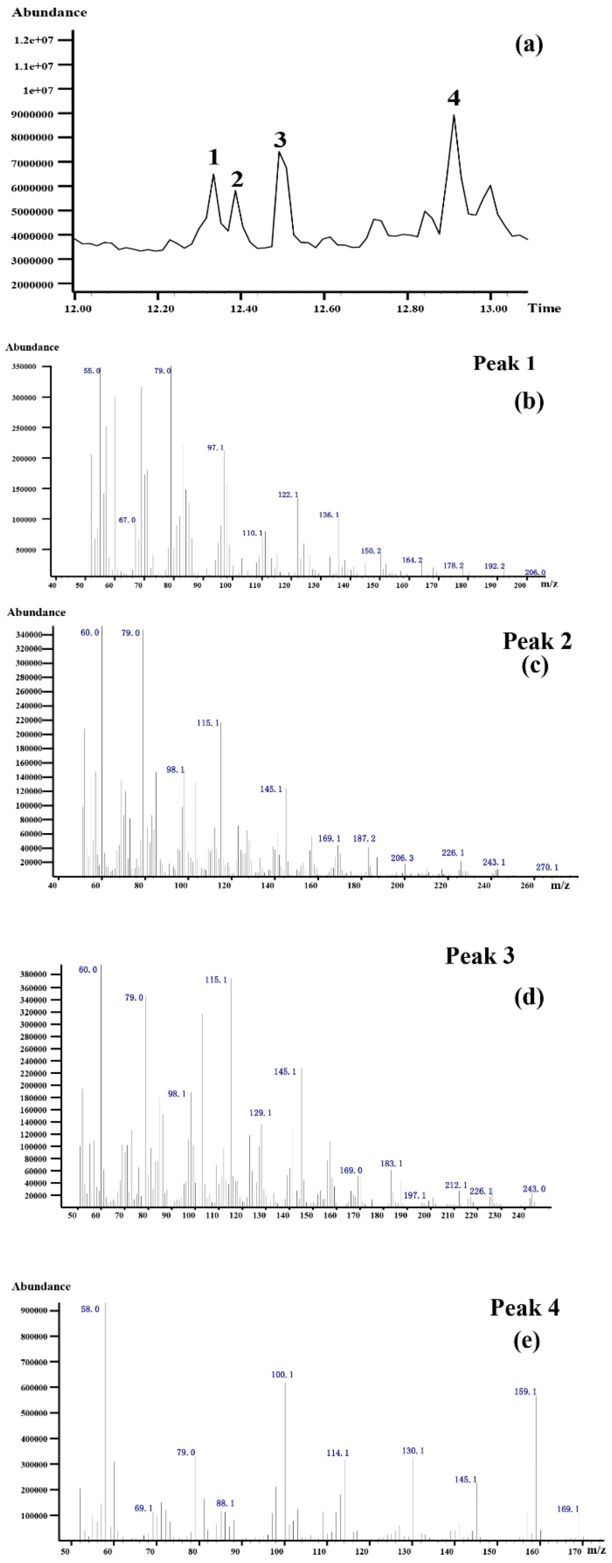

Figure S2. NMR spectrum of SGP-1-1 (a.^{1}H b.^{13}C c. ^{1}H- ^{1}H COSY d. ^{1}C- ^{1}H HSQC).

(a)

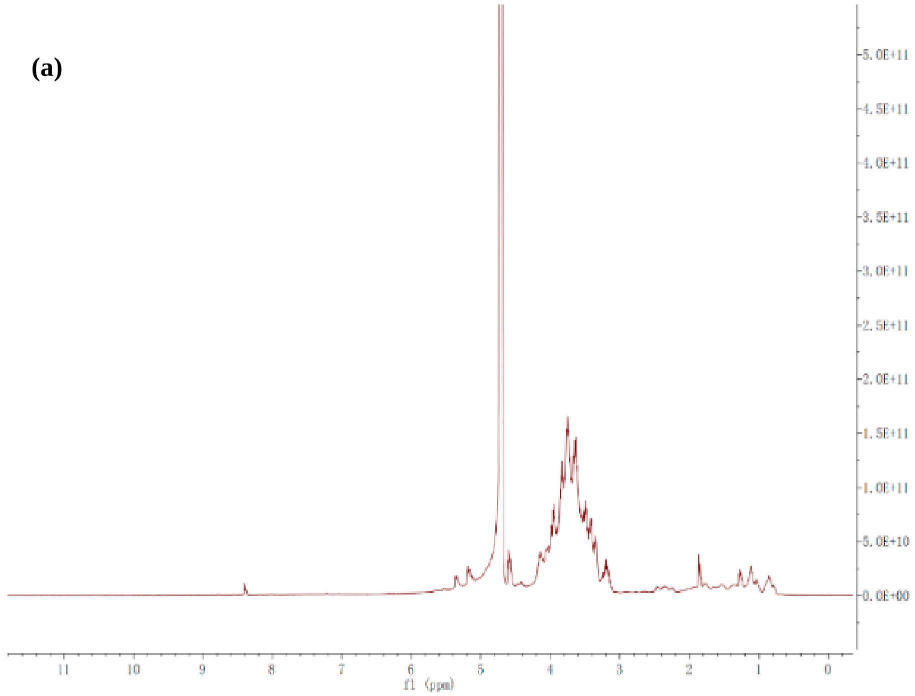

(b)

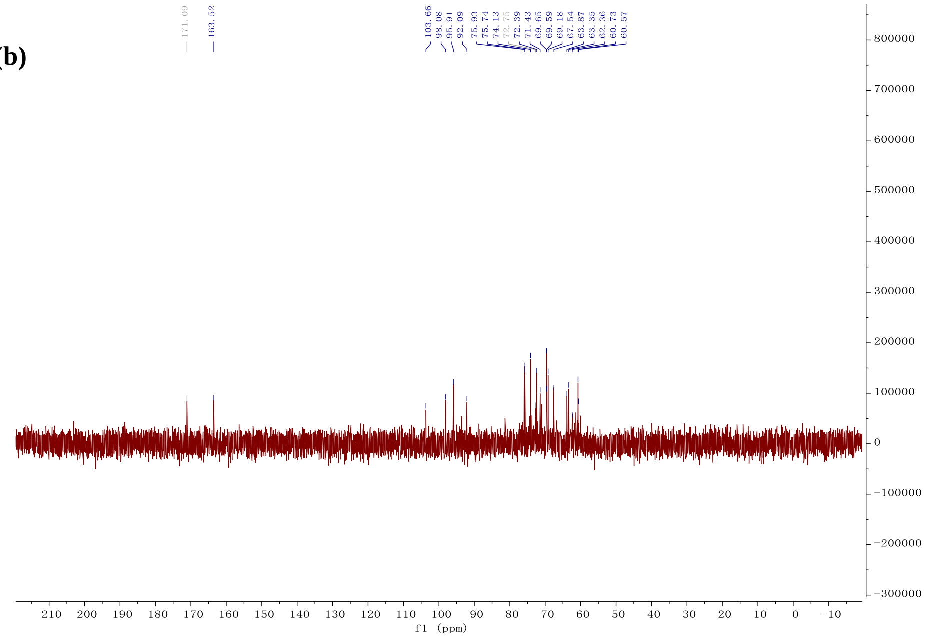

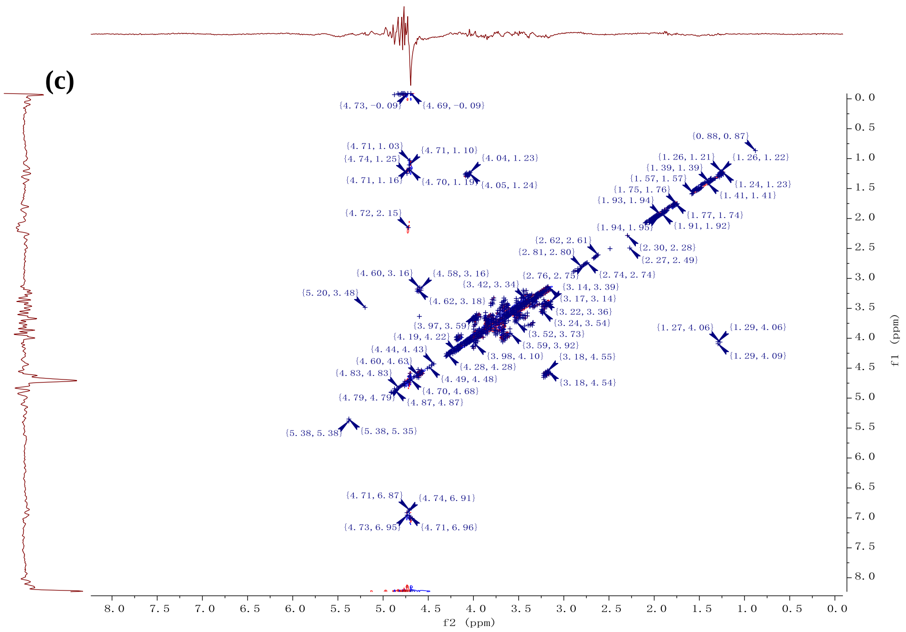
(c)
f2 (ppm)
f1 (ppm)

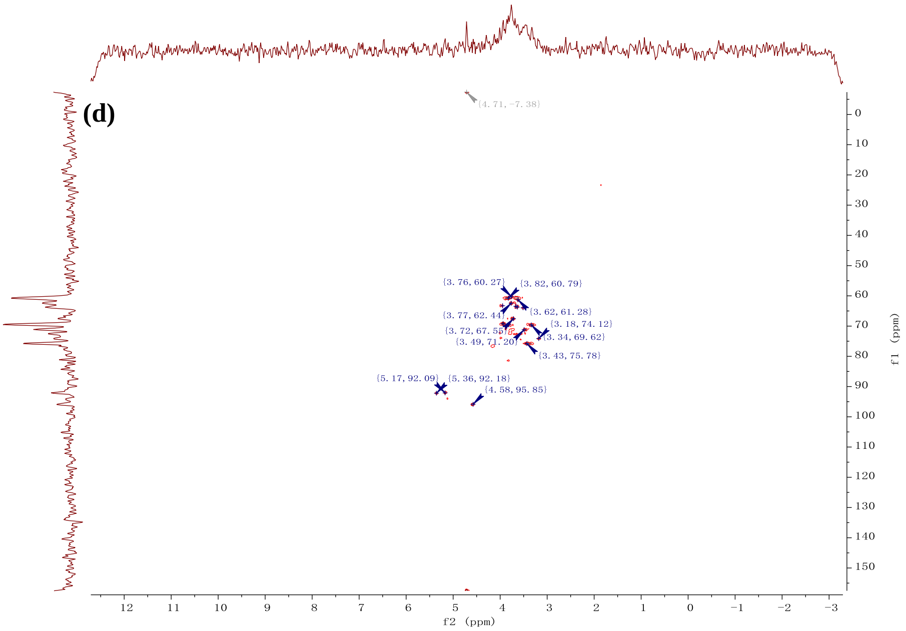
(d)
f2 (ppm)
f1 (ppm)
